# Environmental Risks and Children’s Health in a Mayan Community from Southeast of Mexico

**DOI:** 10.29024/aogh.917

**Published:** 2018-07-27

**Authors:** Hugo Ramírez-Hernández, Javier Perera-Rios, Fernando May-Euán, Gloria Uicab-Pool, Gaspar Peniche-Lara, Norma Pérez-Herrera

**Affiliations:** 1 Unidad Interinstitucional de Investigación Clínica y Epidemiológica, Facultad de Medicina, Universidad Autónoma de Yucatán. Mérida, Yucatán, MX; 2 Facultad de Enfermería, Universidad Autónoma de Yucatán, Mérida, Yucatán, MX

## Abstract

**Background:**

The World Health Organization (WHO) estimates that 25% of global morbidity and one-third of childhood morbidity may be attributable to environment. Low and high-income countries displayed different environmental risks. Mexico demonstrates the necessity for creating a national environmental health program. In southeastern Mexico, Tixméhuac, is a Mayan community with a high marginalization degree and social backwardness. The main childhood morbidities are acute and chronic diseases.

**Objective:**

The aim of the study was to recognize environmental risks for children’s health in Tixméhauc, Yucatan State.

**Methods:**

A total of one hundred children under five years old participated. To identify the environmental risks at home, items from the Green Sheet Guidance (WHO), Salamanca General Survey and Environmental Clinical History were used. To know the prevalence of respiratory diseases and asthma, the International Study of Asthma and Allergies in Children (ISAAC) survey was used. Potentially hazardous sites were identified partially using the Methodology for Identification and Evaluation of Health Hazards in Contaminated Sites from the Pan American Health Organization (PAHO).

**Findings:**

The low stature of, malnutrition, presence of wheezing and asthma symptoms in children were higher than expected. The suspected cases of parasitosis and vector-borne disease occurred in 50% of the children. Indoor air quality perception was associated with respiratory pathology history; housing quality was related to suspected cases of vector-borne diseases; drinking water quality was linked to suspected cases of parasitosis. Risk areas in the community include agricultural activity, which has led to deposits of empty containers of agrochemicals and electronic waste among solid waste dump.

**Conclusion:**

This study presents observed environmental risks to children in a low development country and in developing countries. The community has a low perception of the environmental risk. The need for public health programs reducing risks to children’s environmental health is imperative.

## Introduction

The World Health Organization (WHO) has estimated that approximately 23% of global deaths are attributable to modifiable or preventable environmental factors; in children under the age of 5, this percentage reaches up to 38% [1]. The number of studies reporting evidence of the relationship between health status and environmental factors are increasing. Due to this, WHO implemented the Global Plan of Action for Children’s Health and the Environment, which includes the proposal Making the Difference: Indicators to Improve Children’s Environmental Health [2,3].

Landrigan et al. [4] analyzed the patterns of environmental exposure and disease among low-income countries (LICs), middle-income countries (MICs) and high-income countries (HICs). They observed that air and water pollution have carried the main environmental risks for diseases in LICs and MICs. In the upper middle income countries and HICs, where the problems of water pollution were controlled, the environmental risk factors of concern to children’s health were air quality and exposure to toxic chemicals, pesticides, heavy metals and built environments. So, in countries experiencing this epidemiological transition or change in disease patterns, they must deal simultaneously with both epidemics: infectious and chronic [2].

Globally, the five leading causes of death and illness in children under five years old are respiratory diseases, vector-borne diseases, diarrheal diseases, physical injuries and perinatal diseases [3]. The Pan American Health Organization (PAHO) reported that in the Americas region nearly one thousand children under the age of five die annually due to environmental, physical, chemical and biological threats [5]. The main cause of environment-related mortality in children under fourteen years old in Latin America is respiratory disease [2].

Although Mexico has made progress in children’s environmental health, it requires a national environmental health program to prevent and mitigate the risks and damages to health generated by environmental degradation and pollution [6]. In 2002, the first diagnosis of environmental and occupational health was made in Mexico. It reported states with risks of contamination by metals (mercury, lead, manganese), pesticides, toxins and volcanic emissions and most presented hazardous waste problems. It also reported entities with the highest number of asthma cases, including Yucatan state, whose morbidity rate was higher than the national rate [7]. Subsequently, Cabañas et al. reported in 2012 nearly four thousand potential sources of hazardous waste in Yucatan, with 73.4% concentrated in the ten most populous municipalities in the state [8]. The objective of the study was to identify the risks to children’s health and to know the main health problems of children from Tixméhuac, an ancestry Mayan community from Southeastern Mexico. It’s the community in which we started to show this panorama of Yucatan state.

## Methods

Tixméhuac municipality is located in the south of Yucatán. Karstic ground in this region allows subterranean water to be vulnerable to toxic contamination. The land use is mainly residential, agricultural and livestock. The main economic activity is agriculture, with a moderate specialization in cyclic crops, most of which are located at the periphery of the municipality [9]. Inadequate disposal of empty bottles and uncontrolled use of agrochemicals are significant sources of pesticide contamination.

### Study population

The National Institute of Statistics and Geography reports Tixméhuac has a total population of 4,746, of which 34.1% is between 0 and 14 years old and more than 75% speak in the Mayan language. Tixméhuac is a municipality with a high degree of marginalization and an average social backwardness, where 81% of the population lives in conditions of poverty, with 43% in extreme poverty. Most of the population over 15 years old does not exceed five years of schooling, and 25% of the adult population is illiterate. Between 25% and 50% of households have electricity and piped potable water [9].

Main morbidities reported in 2014 and 2015 for the population under 14 years old were acute respiratory infections and acute diarrheal diseases, with suspected cases of dengue and chikungunya virus and hepatitis [10].

### Environmental clinic history

We include 100 children under 5 years old who were born and live in Tixméhuac municipality and are included in the Niño Sano (healthy kid) program, a government program that provides universal care to children in Mexico, monitoring their growth and development as well as other measures of primary care.

After receiving informed consent from parents or tutors, the survey was conducted through an interview. Questions about general characteristics, weight and height were taken from the clinic history and compared with charts showing the expected average height and weight for a child of that age [11].

To know the prevalence in respiratory diseases and asthma by clinical data we included questions from International Study of Asthma and Allergies in Children (ISAAC) [12] phase one. To identify environmental risks in the home, a structured questionnaire was created with items obtained from the Hoja Verde from WHO [13]. Some of the questions in the WHO environmental clinic history were adapted, focusing on tutors’ perceptions of environmental risk given the difficulty in the measuring. Some questions from the SALAMANCA study survey and from the Environmental Health History survey were included [14].

### Environmental risk in the community

A preliminary list of potentially contaminated sites was used to identify potentially hazardous sites in the study area, implementing phase 1 and phase 2 from the PAHO methodology for identification and risk assessment for contaminated sites [15].

### Statistical analysis

Data from interviews were collected using STATA software v10 (STATA, Corp.), in which we performed the descriptive statistical analysis of variables. Epi Info™ v 3.5.4 were used to obtain Xi^2^ and Fisher tests to evaluate the association between children’s health problems and environmental characteristics.

## Results

### Environmental clinic history

Table 1 shows the characteristics of the study population. The mean age was 2.1 years, 33% had sizes below the 15th percentile and 21% of the children showed some signs of malnutrition.

**Table 1:** Study population characteristics.

Parameter	%

**Age (years)**	2 (0–4)^a^
**Sex**	
Male	55
Female	45
**Size**	
Low	14
Slightly low	19
Normal	64
Slightly high	2
High	1
**Nutritional Status**	
Severe malnutrition	0
Moderate malnutrition	9
Mild malnutrition	12
Healthy weight	75
Overweight	3
Obesity	1

^a^ Median (range).

A family history of respiratory pathology was identified in 25% of the children, while 16% had a clinical history: asthma was the most frequent (11%). Wheezing was reported in 27% of the population, while 22% had wheezing in the last 12 months. Suspected parasitic cases and vector borne diseases were observed in 45% and 54% of the children, respectively (Table 2).

**Table 2:** Health problems in children.

Outcome	%

**Family history of the respiratory pathology**^a^	
Asthma	20
Allergic rhinitis	16
Bronchitis	2
Pulmonary emphysema	1
Tuberculosis	1
**Personal history of respiratory or allergic pathology**^a^	
Asthma	11
Bronchitis or bronchiolitis	7
Pneumonia or bronchopneumonía	5
Dermatitis	1
Allergic rhinitis	1
Tuberculosis	0
**Wheezing presence**	27
**Presence of wheezing in the last 12 months**	22
**Number of episodes of wheezing in the last 12 months**	
1–3	12
4–12	10
>12	0
**Nocturnal wheezing**	
None	92
<1 per week	7
>1 per week	1
**Wheezes that make speech difficult**	6
**Wheezing related to physical activity**	4
**Night cough**	16
**Suspected vector-borne disease**	54
**Suspected parasitosis**	45

^a^ Some patients present 2 or more antecedents simultaneously.

At the children’s homes, 50% of households had contaminated outdoor air, while 97% had contaminated indoor air (Table 3).

**Table 3:** Environmental characteristics of housing where children live.

Characteristic	%

**Perception of food quality**	
Appropiate	19
Doubtful	81
**Outdoor air quality perception**	
Clean	50
Contaminated	50
**Indoor air quality perception**	
Clean	3
Contaminated	97
**Drinking water quality perception**	
Drinking water	47
Median	53
**Other use water quality perception**	
Drinking water	33
Median	67
**Chemical exposure perception**	
Low risk	78
Medium risk	22
**Geographic area**	
Low risk	96
Medium risk	4
**Adequate quality of housing construction**	10
**Adequate ground/floor**	80
**Adequate excreta disposition**	40
**Adequate trash disposition**	16
**Noise**	
Low	96
Medium	4
**Vehicular traffic**	
Low	99
Medium	1

Table 4 shows other environmental problems within homes: 74% of households reported overcrowding, 91% was cooking with firewood and 80% were using pesticides.

**Table 4:** Housing environmental problems.

Characteristic	%

**Overcrowding**	
<3 people sleeping in the same room	26
>3 people sleeping in the same room	74
**Biomass consumption**	
>5 times per week	91
<5 times per week	1
<1 times per week	0
<1 times per month	3
None	5
**Smoking**	28
**Use of pesticides**	80
Intradomiciliary	15
In animals	1
Own crop	54
Neighboring crop	10
**Accidents**	23
Car accident	0
Burn accident	7
Chemical accident	3
Food poisoning	6
Extreme temperatures	4
Poisonous animal	7

On the other hand, most urbanization services were available to almost all the community: electricity (100%), water supply (100%), health center (99%), public transportation (96%) and public lighting (96%). Communication was reported by 45% of respondents, while waste collection and final disposal of solid wastes presented less frequently (31% and 33%). Local treatment plant services and sewage network were nonexistent.

### Exposure and disease

Table 5 shows the association between respiratory-type health problems and household environmental conditions. The history of respiratory pathology showed a marginal association with indoor air quality. Table 6 shows that the suspicion of vector-borne disease was associated with the quality of construction of the house. The suspected cases of parasitosis showed an association with the quality of construction of the house, the quality of drinking water, the type of soil and excreta management.

**Table 5:** Association between respiratory problems and environmental house conditions.

Environmental characteristic	Category	Respiratory pathology antecedent		Wheezing presence		Wheezing in the last 12 months	

		Present	Absent	Present	Absent	Present	Absent

**Indoor air quality perception**	Contaminated	14	83	25	72	20	77
	Clean	2	1	2	1	2	1
p*		0.065		0.176		0.120	

**Overcrowding**	Present	11	63	20	54	15	59
	Absent	5	21	7	19	7	19
p*		0.756		0.991		0.483	

**Use of firewood**	Present	14	79	25	70	20	75
	Absent	2	5	2	3	2	3
p*		0.310		0.410		0.302	

**Burning trash**	Present	15	82	26	71	21	76
	Absent	1	2	1	2	1	2
p*		0.410		0.615		0.529	

* Exact Fisher test applied.

**Table 6:** Association between suspected case of vector-borne disease and suspected case of parasitosis with environmental house conditions.

Environmental characteristics	Category	Suspected case of vector-borne disease		Suspected case of parasitosis	

		Present	Absent	Present	Absent

**Quality construction of housing perception**	Inadequate	53	37	45	45
	Adequate	1	9	0	10
p*		0.005		0.002	

**Quality of drinking water perception**	Contaminated	33	34	29	25
	Potable	21	12	16	30
p*		0.176		0.05	

**Overcrowding**	Present	41	33	37	37
	Absent	13	13	8	18
p*		0.635		0.091	

**Quality of food perception**	Adequate	42	12	6	13
	Medium	39	7	39	42
p*		0.394		0.193	

**Soil**	Inadequate	13	7	14	6
	Adequate	41	39	31	49
p*		0.272		0.012	

**Excreta management**	Inadequate	35	35	34	26
	Adequate	19	21	11	29
p*		0.801		0.004	

* Exact Fisher test applied.

### Environmental risks in the community

Five potentially dangerous sites were identified in Tixméhuac. None of the sites were classified as an environmental and public health emergency. There were three sites in the category of environmental risk and public health: the orchard, the municipal landfill, and the agrochemical store.

The orchard is where fruits are grown for human consumption. Agrochemicals are stored at the entrance in an improvised container made with wooden sticks and cardboard sheets. We estimate that more than 100 containers of agrochemicals were deposited without any special precautions.

The municipal landfill is the final disposition site of municipal solid waste, which is stacked in the open air directly on the ground without any special care. Periodically, the waste is burned to free up space. There is a wide variety of residue found: metal cans, crystals, pet bottles, electronic waste (multiple TVs, cathode ray tubes, etc.) and mechanical waste.

The agrochemical store distributes agrochemicals at the municipal level. It handles several products, including pesticides and fertilizers. Where products are stored in a separate room from the home, no odors are detected that suggest contamination or open or half-used containers.

We found two sites in the category of minimum environmental and public health risk: the bakery and the aguada.

The bakery has a wood oven that releases smoke to the outside. In addition, poultry slaughtering activities are frequently carried out on site. There is concern for the unhygienic conditions in which poultry activities are carried out.

The aguada is where waste is deposited from a pig and poultry breeding area, whose entrance is close to the public road and serves as a garbage deposit. The site has social vulnerability and directly impacts soil, surface water and groundwater.

## Discussion

The main health problems observed in Tixméhuac children were suspicious cases of vector-borne diseases and parasitic diseases, which appeared in almost half of the population, followed by a clinical history of respiratory pathology. Vector-borne diseases are endemic to Yucatan, where dengue virus is the main problem [16]. Other vector-borne diseases transmitted by ticks and fleas, like rickettsiosis, have been emerging in Yucatan since 2001 and mainly infecting children [17].

Knowledge of vector-borne diseases is important in Yucatan and other tropical regions. Medics must be informed about their presence and specific signs and symptoms [18]. Communities must be informed about infectious diseases other than dengue fever. Public health programs should design programs that include tick and flea prevention strategies. Patients and family members should be treated for parasitosis. Although it is true that the diagnosis is not established only by symptomatology or sick relatives, its frequency is similar to the 49.1% reported by the Sistema Nacional de Vigilancia Epidemiológica (National epidemiological surveillance system) [19]. Regarding respiratory diseases, the frequency of children with wheezing is similar to results from Aguilar et al. in 2009 for children and adolescents (26.6%) living in Merida, the capital and urban area of Yucatan. Although the frequency of children with wheezing in the last 12 months is double that reported in the same study [20].

This profile of diseases has traditionally been related to environmental conditions. Landrigan et al. [4] noted that global exposure patterns and health problems are changing and vary across countries. Poor water and indoor air quality are environmental problems related to infectious-parasitic and respiratory diseases in low-income and lower middle-income countries [4]. Mexico is in the stage of epidemiological transition and with heterogeneity in the regions that comprise it. The southern region has been reported as having a higher burden of disease and mortality than the other regions [21].

Nutritional status is an important indicator in health, especially in child populations. This study found the low frequency (33%) is twice as high as that reported by ENSANUT in 2012 for Yucatán (15.8%). Low weight was also observed with a markedly greater frequency (21%) than that reported by ENSANUT (1.4%). The quality of the food was classified as doubtful in its preparation in a wide proportion (81%). In many cases, there is no refrigeration or adequate preservation and storage methods. There are also inadequate hygiene methods when preparing food. Couple this with the fact that Tixméhuac is a municipality with a high degree of marginalization and an average social gap, where 81% of the population lives in poverty, where 43% lives in extreme poverty, where education levels are low [9] and where 78% of the population has a low knowledge of toxic exposure, and you get an increase in the vulnerability of children to environmental hazards.

A study in developed countries using the WHO green leaf determined that 24% of parents were concerned about the environmental health of their children and 23% were aware of some environmental risk. In this study, the environmental characteristics were low risk in most of the items evaluated. The indoor air was of good quality in 73% of the cases, while outdoor air was of medium quality in 80% of the cases [22].

In our study, the perception of indoor and outdoor air quality was very different. Clean outdoor air was reported by half the respondents due to the scarcity of factories that pollute the air. Indoor air quality was perceived as predominantly contaminated (97%) due to the use of wood as an indoor fuel in practically all households; although ENSANUT estimates it in 33% of homes [23]. In Mexico, 90% of the rural population uses solid fuels (firewood) as the main domestic energy, [24] and it is in these homes that the highest levels of pollution have been reached, with values of PM_25_ up to 1000 µg/m^3^ [25].

In this study, we identify risks to children’s health due to conditions in which they live. The quality of house construction was predominantly inadequate (90%), usually due to the lack of one or more urbanization services. In addition, overcrowding occurs in one out of every three homes, and the disposition of excreta was observed in less than half of them. Although the garbage collection service is available to almost the entire community, less than 20% of households opt for it because the practice of burning garbage in the home persists. At the national level, less than 3% of solid waste is managed in open pit dumps, which is a situation that is predominant in Tixméhuac [7]. The use of pesticides in the home is a common practice in this rural community, although the knowledge of their chemical risks is low. Finally, there was a higher frequency of documented accidents in children under 5 years of age (23%) compared to that reported by ENSANUT (4.6%), [23] although none resulted in serious complications. When comparing our results with those obtained in developed countries, there is a marked difference, except for outdoor air quality. All the indicators were lower in developed countries, including a greater concern about environmental risks [14].

The history of respiratory pathology in children was marginally associated with the perception of indoor air pollution, which is probably explained by the use of firewood as fuel. It is well known that burning solid wastes and agricultural wastes, as well as the use of solid fuels such as coal and biomass for cooking and heating, are an air pollution problem of great concern. Globally, an estimated 3 billion people are engaged in these practices, resulting in high levels of indoor air pollution. In poorly ventilated homes, fine particle levels may exceed acceptable levels by more than 100 times, which is a risk mainly for women and children who spend more time in the home [26]. Studies have shown that exposure to biomass smoke is a predisposing factor to chronic obstructive pulmonary disease. Pathogenic mechanisms are based on increased markers of pulmonary and systemic inflammation, as well as increased formation of reactive species of oxygen, which damage macromolecules like DNA [27].

Suspected cases of vector-borne disease were associated with quality of housing construction. They lack structures that protect against vector entry into the home, which, in addition to overcrowding and inadequate disposal, contribute to high vector-borne disease frequency. The suspicion of parasitosis had a greater environmental burden and was associated with the quality of construction of the house, water quality, soil type and excreta management. A study conducted in India showed a similar association between the prevalence of parasitic disease and the management of excreta [28].

In addition to these threats to children’s health observed in homes, we identify potentially dangerous sites in the community. In the dump, regular burning of garbage represents a source of air pollution. It is estimated that cities annually generate 1.3 trillion tons of garbage. By 2025, that is expected to increase to 2.2 trillion tons. This represents another global environmental health challenge. Inadequate garbage collection and disposal favors the proliferation of vectors carrying diseases of public health importance [29]. Hazardous waste, such as electronic waste and cathode ray tubes, which may contain heavy metals such as lead, are also found at this site and require proper handling and disposal. In the site identified as the orchard, an inadequate storage of pesticide and fertilizer containers was observed [30]. Considering that the soil of Yucatan is karst type, leaching processes in the rainy season represent a risk of contamination of the water table, the only source of water supply for domestic consumption. Studies conducted at sites where agrochemical containers have been deposited outdoors or buried have shown, the persistence of contaminants in the soil and wells [30]. Landrigan et al. [4] describe air pollution, pesticides and heavy metals as among the main risks for children in HIC.

Our results show that in the community of Tixméhuac, Yucatán, children are developing in an environment that combines classic threats from developing countries, such as inadequate housing conditions, use of fuelwood and burning of garbage, which compromise air quality. Hazards in the community, such as the garbage dump and the deposit of pesticide containers, endanger the quality of the water. This community also faces environmental risks inherent in developed countries, such as the use of pesticides in the home and exposure to metals and other toxic substances in electrical and electronic waste. All this, combined with poverty, social backwardness and low education levels are social determinants that increase the vulnerability of children’s health.

Landrigan et al. [4] conclude that the problem of pollution is growing globally and is of such magnitude that a global response like that applied to controlling AIDS, tuberculosis and malaria will be needed to save the lives of millions of people. Laborde et al. [2] suggest that to control environmental threats to the health of children in Latin America, WHO and PAHO should focus on the most serious threats to a greater number of children: indoor and outdoor air pollution, water pollution and toxic substances. Riojas et al. [6] mention that a cross-sectoral perspective is required in Mexico to address environmental health problems in an integrated environmental health surveillance system. They propose creating a national environmental health program and updating national and regional diagnoses while strengthening teaching and research.

## Conclusion

Our results show, for the first time, a diagnosis of children’s environmental health in a Mayan descent community in Yucatan. We observed threats to children’s health related to the characteristics of homes, and these were associated with vector-borne diseases. Suspected cases of parasitosis had an important environmental burden determined by quality of housing construction, water quality, soil type and excreta management. The management of these was adequate in half of the cases. Indoor air perceived as contaminated due to the use of firewood as the main source of fuel for cooking and probably due to the burning of garbage was weakly associated with a history of respiratory disease. The results show a mix of traditional and emerging threats with global distribution. We consider that a policy is required from the integrative vision of public health to articulate the different sectors for improving the health and environment conditions of Tixméhuac and similar communities.
